# Bile Duct Injuries in Connection With 2500 Laparoscopic Cholecystectomies

**DOI:** 10.1155/DTE.4.55

**Published:** 1997

**Authors:** P. Schmidt, P. Ezer, A. Antal

**Affiliations:** 2nd Department of Surgery Pécs University Medical School Irgalmasok u. 1. Pécs H-7621 Hungary

## Abstract

*Background*: Laparoscopic cholecystectomy (LC) is taking the place of an effective and tested procedure in surgery, therefore it must not be inferior to the standard modality in any aspect. Some complications specific to the technique, however, are severe and complication rate seem to be higher than in standard open surgery.

*Methods*: In this paper the authors report their guiding principles in applying LC and methods of treatment, and describe the bile duct injuries of 2500 LCs accomplished during the past 4.5 years.

*Results*: Seventeen ductal injuries occurred in the whole series, which means an overall incidence rate of 0.68%. Data obtained in the last period, however, show a decrease down to 0.14%. Following ductal injuries six ductal strictures became clinically apparent. The various complications of these injuries caused the death of two patients.

*Conclusions*: The great number of intraoperatively undetected injuries, many of them arising not due to technical difficulties, suggest the possibility of an injury caused by electric current. Depending on the type of injury direct suture, T-tube drainage or biliodigestive anastomosis can equally be effective. Long established practice and experience can help reduce the occurrence of complications to the level in standard open surgery.


*Diagnostic and Therapeutic Endoscopy*, Vol. 4, pp. 55–60
Reprints available directly from the publisher
Photocopying permitted by license only

© 1997 OPA (Overseas Publishers Association)
Amsterdam B.V. Published under license
in The Netherlands under the Harwood Academic
Publishers imprint, part of The Gordon and
Breach Publishing Group.
Printed in Singapore.

### Bile Duct Injuries in Connection with 2500 Laparoscopic Cholecystectomies

P. SCHMIDT*, P. EZER and A. ANTAL

*2nd Department of Surgery, Pécs University Medical School, H-7621 Pécs, Irgalmasok u. 1., Hungary*

*(Received 29 January 1997; Revised 24 March 1997; In final form 1 May 1997)*

**_Background_: Laparoscopic cholecystectomy (LC) is taking the place of an effective and tested procedure in surgery, therefore it must not be inferior to the standard modality in any aspect. Some complications specific to the technique, however, are severe and complication rate seem to be higher than in standard open surgery.**

**_Methods_: In this paper the authors report their guiding principles in applying LC and methods of treatment, and describe the bile duct injuries of 2500 LCs accomplished during the past 4.5 years.**

**_Results_: Seventeen ductal injuries occurred in the whole series, which means an overall incidence rate of 0.68%. Data obtained in the last period, however, show a decrease down to 0.14%. Following ductal injuries six ductal strictures became clinically apparent. The various complications of these injuries caused the death of two patients.**

**_Conclusions_: The great number of intraoperatively undetected injuries, many of them arising not due to technical difficulties, suggest the possibility of an injury caused by electric current. Depending on the type of injury direct suture, T-tube drainage or biliodigestive anastomosis can equally be effective. Long established practice and experience can help reduce the occurrence of complications to the level in standard open surgery.**

*Keywords:* Bile duct injuries, Cholelithiasis, Complications, Laparoscopic cholecystectomy

### INTRODUCTION

In the past few years LC has become the procedure of choice for the management of biliary stone disease. Specific or non-specific complications are said to occur in 3–7% of cases (Berry *et al.*, 1994; Schlumpf *et al.*, 1994), which compares LC to standard open cholecystectomy (The Southern Surgeons Club, 1991). Alongside with this figure, however, it must be noted that some complications may be more common after LC than after the standard open procedure, which is a basis for concern. For a new surgical method to gain acceptance is always difficult but the introduction of LC raises an ethical dilemma as well. Open cholecystectomy has been an effective, tried

* Corresponding author. Tel.: 36/72/311-785. Fax: 36/72/324-358.

and tested method; the newer treatment must not be inferior to the old one in any aspect.

In reviewing literature, it is obvious that results in relationship to applying LC have been improving. In order to minimize the occurrence of avoidable complications, it is important to draw conclusions from the fallacies of this surgical technique.

The aim of the present paper is to retrospectively assess bile duct injuries in order to reduce their occurrence and assist their early detection and successful management.

### MATERIALS AND METHODS

In Hungary the first LC was performed at our department in December 1990. Since then, until May 1995, we have accomplished altogether 2500 LCs. The minimum follow-up of these patients was 18 months. The first 200 operations were performed by four surgeons, the next 700 by six and the rest by 16. The age of the patients ranged from 6 to 85 years.

In the first six months LC was applied only in simple cases. Later the application of LC was gradually extended. Today neither a history of abdominal surgery nor acute cholecystitis is a contraindication. The last 700 LCs performed during the last year accounted for 95% of the total number of operations for gallstones. Concurrently, conversion rate in complicated cases dropped from an initial 29.6% to 13.8%.

In our series a four port technique was used and the gallbladder was removed via the epigastric port. No drainage was applied during the first 200 operations. Because of some late detected ductal injuries, in this series, the insertion of a subhepatic drain at the closure of operation has become the normal practice.

Intraoperative cholangiography was obtained only on a selective basis, in the beginning primarily in suspicion of choledochal stones, later in the case of unclear anatomy or for detecting a possible ductal injury. To recognize suspected ductal injuries postoperatively, ultrasonic examination and ERCP were primarily obtained.

Patients were reviewed routinely one month after surgery and later in every year. If any complaints arose, liver function tests, ultrasonic evaluations and ERCP were performed.

### RESULTS

Seventeen ductal injuries occurred in the whole series, which means an overall incidence rate of 0.68%. Data obtained after the starting period, however, show a decrease. In the first 600 cases the rate was 1.66%; in the next 1200 cases 0.50%; and in the last 700 cases 0.14% (Table I). From the 17 ductal injuries 4 were observed intraoperatively, and 13 postoperatively. Intraoperative cholangiography was not performed in either cases.

At the four intraoperatively detected injuries technical diffculties were encountered only in one case. In these cases LC was immediately converted to open surgery, which facilitated recovery without complications; no strictures developed. (The site and type of injuries and the type of surgery are shown in Table II.)

TABLE I Rates of ductal injuries in different consecutive groups of patients

| 600 LCs | 1200 LCs | 700 LCs |
|---|---|---|
| 1.66% | 0.50% | 0.14% |
| Overall incidence: 0.68% | | |

TABLE II Injuries ($n = 4$) detected intraoperatively and their management

| | $n$ |
|---|---|
| Site of injury | |
| Common hepatic duct | 2 |
| Common bile duct | 2 |
| Type of injury | |
| Punctiform | 2 |
| Tangential | 2 |
| Surgery | |
| Suture | 1 |
| T-tube | 3 |

Besides the four intraoperatively noted cases, there were 13 postoperatively detected ductal injuries. Three patients had to be readmitted to hospital 5 to 12 days following surgery; this time no postoperative drain was used. Later drainage of the field of operation became a routinely applied procedure. In the ten further cases it was the ample bile leakage through the drain that directed our attention to the possibility of a ductal injury. Among the clinical signs biliary fistula and severe local pain were the most frequent symptoms (Table III). Alongside with the clinical picture, abdominal ultrasonic evaluation was obtained in each case and ERCP in 6 to facilitate diagnosis. In three cases emergent surgical intervention was necessary due to bile peritonitis. Therefore ERCP was not performed in these cases. In four earlier cases, obtaining ERCP had not been considered yet. All the 6 ERCPs yielded diagnosis and accurately localised the ductal injury. Besides ERCP, PTC and HIDA scan in two cases, and CT scan in one case were obtained (Table IV).

In these cases, according to surgical records about LCs, technical difficulties were encountered only in five cases, whereas 8 operations were accomplished without any difficulties. (The site and type of injuries are shown in Table V.)

Two punctiform ductal injuries were repaired with two sutures, and an uneventful recovery followed. In four cases T-tube drainage of the site of the injury facilitated recovery without complications.

TABLE III Occurrence of symptoms in 13 ductal injuries detected postoperatively

| Symptoms | *n* |
|---|---|
| Bile peritonitis | 4 |
| Icterus | 5 |
| Discomfort | 6 |
| Nausea, vomiting | 6 |
| Paralytic ileus | 9 |
| Severe local pain | 11 |
| Biliary fistula | 13* |

*Three patients without drainage had subhepatic bile collection.

TABLE IV Diagnostic evaluation obtained in presumed ductal injuries

| Method | *n* |
|---|---|
| Ultrasound | 13 |
| ERCP | 6 |
| PTC | 2 |
| Tc 99m HIDA | 2 |
| CT | 1 |

TABLE V Lesions detected postoperatively ($n = 13$)

| | *n* |
|---|---|
| Site of injury | |
| Right hepatic duct | 3 |
| Common hepatic duct | 8 |
| Common bile duct | 2 |
| Type of injury | |
| Punctiform | 5 |
| Tangential | 6 |
| Transsection | 2 |

In two cases subhepatic biloma was recognized and drained but drainage was of acute help only; a subsequent stricture developed in the site of the injury. Hepatico-jejunostomy was accomplished with success in one case, but in the other case the stricture reappeared. In this latter case, repetition of hepatico-jejunostomy and continuous Voelcker drainage facilitated the patient's recovery. In three cases after removal of the T-tube from the site of the injury a stricture developed. Hepatico-jejunostomy was successfully performed in two cases, whereas in the third case choledochoplasty and prolonged T-tube drainage were the scenario of managing the stricture. In one fatal case, after transsection, bilio-biliary reconstruction and Voelcker drainage preceded the development of a stricture. This was followed by two hepatico-jejunostomies, indicated by stricture and anastomosis insufficiency. Following the exposure of local abscesses the patient died of sepsis. In one case the effectiveness of the T-tube placed in the punctiform injury could not be evaluated because the readmitted and reoperated patient died on the day of reoperation, that is, on the 11th day after initial surgery (Table VI).

TABLE VI Management and outcome of the 13 postoperatively detected ductal injuries

| 1st operation | Outcome | 2nd operation | Outcome | 3rd operation | Outcome |
|---|---|---|---|---|---|
| Suture | Recovery | | | | |
| Suture | Recovery | | | | |
| T-tube | Recovery | | | | |
| T-tube | Recovery | | | | |
| T-tube | Recovery | | | | |
| T-tube | Recovery | | | | |
| T-tube | Death | | | | |
| T-tube | Stricture | H-J | Recovery | | |
| T-tube | Stricture | H-J | Recovery | | |
| T-tube | Stricture | Ch-plasty | Recovery | | |
| Drainage | Stricture | H-J | Recovery | | |
| Drainage | Stricture | H-J | Stricture | H-J | Recovery |
| B-B-anast. | Stricture | H-J | Anast. insuff. | H-J | Sepsis, death |

B-B anast = bilio-biliary anastomosis; H-J = hepatico-jejunostomy; Ch-plasty = choledochoplasty.

There were altogether six patients who developed strictures in the site of the injury in the first few postoperative weeks. Of the six strictures, one occurred after bilio-biliary anastomosis, and the other five developed following drainage or T-tube drainage. In two cases tertiary surgery was necessary.

The follow-up of the patients undergone reconstructional surgery ranged between 30 and 65 months. No further strictures were noted after the aforementioned surgical interventions although in four cases liver function tests were mildly elevated.

### DISCUSSION

According to large series, the incidence of ductal injuries is 0.3–0.7% (Wayand *et al.*, 1992; Schlumpf *et al.*, 1994) but rates of 0.1%, 2.0% and even 2.9% have also been reported (Kerin and Gorey, 1994; Macintyre and Wilson, 1993), whereas the incidence of ductal injuries in open surgery is 0.2% (Hunter, 1991). The rates in dated reports are usually higher than those in more recent ones. The overall incidence of ductal injuries among our patients was 0.68% but data obtained after the starting period show a decrease: from 1.66%, at the beginning, to 0.14%, in the last period. A high percentage of such injuries remain undetected at the time of intervention (Schlumpf *et al.*, 1994) even when the operations are presupposedly "easy" (Adams *et al.*, 1993). Our data also support this observation.

To detect a ductal injury postoperatively as soon as possible, drainage is necessary. Some authors applying selective drainage, report on delayed detected injuries within 3–8 days (Brooks *et al.*, 1993). Because of some ductal injuries observed delayed, we always drain the field of operation in order to obtain early diagnosis. Temperature, severe pain below the right costal arch or peritonitis are alarming clinical signs. ERCP seems to be the most reliable diagnostic procedure in our series.

Some authors believe there is a relationship between the injury and current conduction through the opened cystic duct to the junction of the choledochal duct (The Southern Surgeons Club, 1991). It is believed that, because of high tissue resistance, conversion of electrical to thermal energy occurs with subsequent thermal burn. Naturally, surgical instruments themselves may cause burn through direct contact (Berry *et al.*, 1994; The Southern Surgeons Club, 1991). Such thermal damages by a surgical device may have been the cause of the punctiform injuries and some of the tangential injuries we encountered. In the wake of these serious injuries the use of electrocautery in dissecting in Calot's triangle has become limited, which might also contribute to the decrease in the rate of injuries.

The role of intraoperative cholangiography in preventing ductal injuries is a debated issue. Berci *et al.* (1991) considers routine intraoperative cholangiography a successful means of preventing ductal injuries, whereas other authors (Macintyre and Wilson, 1993) argue this. Nevertheless, it is now evident that intraoperative cholangiography provides reliable information regarding injuries (Raute and Schaupp, 1988). It seems quite obvious that intraoperative cholangiography could have reduced the number of our lately detected injuries. Because of the failure rates of 10–20%, fake positive and negative results, furthermore, costeffectiveness, and the time consuming nature of intraoperative cholangiography, numerous centres, like ours, are in favour of a policy of selective intraoperative cholangiography. Kerin and Gorey (1994) estimate that routine intraoperative cholangiography increases the number of redundant choledochotomies to a rate approaching 19%.

Current management of injuries is similar to procedures developed back in the era of standard open surgery. Nowadays, hepatico-jejunostomy is the most widespread method. Some authors expressly suggest a two-phase management of the injury: as long as bile peritonitis continues, only drainage should be applied, then reconstruction should follow after 4–6 weeks. This practice allows the omission of further reconstruction, thus offsetting a considerably higher risk of restenosis (Manger *et al.*, 1993). Our experience suggests that small punctiform injuries can be repaired with direct suture. We believe that in the event of minor tangential injury T-tube insertion, otherwise bilio-digestive anastomosis, is the appropriate procedure. Common bile duct strictures developing after corrective surgery are not uncommon. The aetiology of the strictures recognized in our patients may have been too short stenting, 10–14 days, in most of the cases.

Long-term assessment can only be made if follow-up is two years or more; the fact that strictures may be manifested as late as 9–10 years after surgery presents a further problem (Adams *et al.*, 1993). The ultimate conclusions are thus to be drawn.

### CONCLUSIONS

Most of the ductal injuries remained undetected intraoperatively and many of them arose when there were no technical difficulties in performing the operation. This suggests the possibility of an injury caused by electric current.

In case of small punctiform injuries direct sutures can be performed with success.

T-tube insertion is the best procedure in the case of small tangential lack of tissue but in the case of large tissue defect we consider the biliodigestive anastomosis as the most appropriate technique.

The prognosis for injuries detected intraoperatively and managed as soon as possible can be regarded as fairly good. In the cases where the injury is detected delayed, however, the number of complications, following reoperation, seem to be quite frequent.

Long established practice and experience help reduce the number of complications to the level in standard open surgery. The fairly low 0.14% frequency rate of ductal injuries, which had been achieved gradually by the last period of our series, underlines this statement.

### *References*

[1] Berry, S.M., Ose, K.J., Bell, R.H. and Fink, A.S. Thermal injury of the posterior duodenum during laparoscopic cholecystectomy. *Surg. Endosc.* 1994; **8**: 197–200.

[2] Schlumpf, R., Klotz, H.P., Wehrli, H. and Herzog, U. A nation's experience in laparoscopic cholecystectomy. Prospective multicenter analysis of 3722 cases. *Surg. Endosc.* 1994; **8**: 35–41.

[3] The Southern Surgeons Club. A postoperative analysis of 1518 laparoscopic cholecystectomies. *N. Engl. J. Med.* 1991; **324**: 1073–1078.

[4] Wayand, W. and Woisetschlager, R. Aktueller Stand der laparoskopischen Cholecystectomie. *Acta Chir Austriaca* 1992; **24**: 260–262.

[5] Kerin, M.J. and Gorey, T.F. Biliary injuries in the laparoscopic era. *Eur. J. Surg.* 1994; **160**: 195–201.

[6] Macintyre, I.M.C. and Wilson, R.G. Laparoscopic cholecystectomy. *Br. J. Surg.* 1993; **80**: 552–559.
[7] Hunter, J.G. Avoidance of bile duct injury during laparoscopic cholecystectomy. *Am. J. Surg.* 1991; **161**: 71.
[8] Adams, D.B., Borowicz, M.R., Wooton, F.T. and Cunnigham, J.T. Bile duct complications after laparoscopic cholecystectomy. *Surg. Endosc.* 1993; **7**: 7983.
[9] Brooks, D.C., Becker, J.M., Connors, P.J. and Carr-Locke, D.L. Management of bile leaks following laparoscopic cholecystectomy. *Surg. Endosc.* 1993; **7**: 292–295.
[10] Berci, G., Sackier, J.M. and Paz-Partlow, M. Routine or selected intraoperative cholangiography during laparoscopic cholecystectomy? *Am. J. Surg.* 1991; **161**: 355–360.
[11] Raute, M. and Schaupp, W. Iatrogene Schaden an den Gallenwegen infolge Cholecystectomie. Behandlung und Ergebnisse. *Langenbecks Arch. Klin. Chir.* 1988; **373**: 345–354.
[12] Manger, T., Pertschy, J. and Wolff, H. Iatrogene Gallengangslasionen nach laparoskopischer Cholecystectomie. *Min. Invas. Chir.* 1993; **2**: 46–52.

